# Comparative Genomic and Transcriptomic Characterization of the Toxigenic Marine Dinoflagellate *Alexandrium ostenfeldii*

**DOI:** 10.1371/journal.pone.0028012

**Published:** 2011-12-02

**Authors:** Nina Jaeckisch, Ines Yang, Sylke Wohlrab, Gernot Glöckner, Juergen Kroymann, Heiko Vogel, Allan Cembella, Uwe John

**Affiliations:** 1 Alfred Wegener Institute for Polar and Marine Research, Bremerhaven, Germany; 2 Medizinische Hochschule Hannover, Institut für Medizinische Mikrobiologie und Krankenhaushygiene, Hannover, Germany; 3 Berlin Center for Genomics in Biodiversity Research, Berlin, Germany; 4 Institute for Freshwater Ecology and Inland Fisheries, Berlin, Germany; 5 Université Paris-Sud/CNRS, Laboratoire d'Ecologie, Systématique et Evolution, Orsay, France; 6 Max Planck Institute for Chemical Ecology, Jena, Germany; American University in Cairo, Egypt

## Abstract

Many dinoflagellate species are notorious for the toxins they produce and ecological and human health consequences associated with harmful algal blooms (HABs). Dinoflagellates are particularly refractory to genomic analysis due to the enormous genome size, lack of knowledge about their DNA composition and structure, and peculiarities of gene regulation, such as spliced leader (SL) trans-splicing and mRNA transposition mechanisms. *Alexandrium ostenfeldii* is known to produce macrocyclic imine toxins, described as spirolides. We characterized the genome of *A. ostenfeldii* using a combination of transcriptomic data and random genomic clones for comparison with other dinoflagellates, particularly *Alexandrium* species. Examination of SL sequences revealed similar features as in other dinoflagellates, including *Alexandrium* species. SL sequences in decay indicate frequent retro-transposition of mRNA species. This probably contributes to overall genome complexity by generating additional gene copies. Sequencing of several thousand fosmid and bacterial artificial chromosome (BAC) ends yielded a wealth of simple repeats and tandemly repeated longer sequence stretches which we estimated to comprise more than half of the whole genome. Surprisingly, the repeats comprise a very limited set of 79–97 bp sequences; in part the genome is thus a relatively uniform sequence space interrupted by coding sequences. Our genomic sequence survey (GSS) represents the largest genomic data set of a dinoflagellate to date. *Alexandrium ostenfeldii* is a typical dinoflagellate with respect to its transcriptome and mRNA transposition but demonstrates *Alexandrium*-like stop codon usage. The large portion of repetitive sequences and the organization within the genome is in agreement with several other studies on dinoflagellates using different approaches. It remains to be determined whether this unusual composition is directly correlated to the exceptionally genome organization of dinoflagellates with a low amount of histones and histone-like proteins.

## Introduction

Dinoflagellates are important marine primary producers and contribute significantly to the functioning of marine food webs. Nevertheless, many dinoflagellates are also a rich source of marine toxins and can form dense aggregations of cells known as harmful algal blooms (HABs). These dinoflagellates and their toxins pose a threat for fish, wildlife, and also to humans via consumption of contaminated seafood or direct exposure to HABs, particularly in coastal regions throughout the world.

The dinoflagellate genus *Alexandrium* (Halim) Balech contains more neurotoxin-producing members (about 12 described species) than any other described algal genus [1]. One species, *A. ostenfeldii*, is associated with the production of cyclic imine toxins known as spirolides with fast-acting toxicity in mammals [2]. In recent decades the frequency and expansion of toxic *Alexandrium* blooms and related toxic events has increased, as have HABs in general, possibly due to increased scientific awareness of toxic species and their effects, as well as to shifts in environmental regimes associated with climate change, cultural eutrophication, transport of resting cysts either in ship's ballast water or by movement of shellfish stocks from one area to another [3], [4].

The dinoflagellate genome is very special with respect to structure and regulation compared to all other eukaryote genomes (reviewed by [5]). Up to 70% of the genome contains unusual bases with a high degree of methylation, e.g. 12–70% of the thymine is replaced by hydroxymethyluracil, which makes up 4–19% of all bases [6]. Analyses of genomic sequences of dinoflagellate genes revealed that they lack recognizable promoter features like TATA boxes and common eukaryotic transcription factor binding sites [7], [8]. The nucleus of typical dinoflagellates contains chromosomes that are permanently condensed throughout the cell cycle, displaying a liquid crystalline state [9]. Actively transcribed DNA protrudes from the condensed chromosome core in peripheral loops of B- and Z-DNA [10]. Although vegetative cells of *Alexandrium* species are nominally haploid, as is the case for almost all known free-living dinoflagellates, the nucleus can contain up to 200 pg DNA per cell. The nuclear DNA content of *A. ostenfeldii* has been estimated at 115 pg DNA per cell [11]. In dinoflagellates the nuclear DNA is organized into as many as 200 morphologically indistinguishable chromosomes that are attached to the nuclear envelope [12]. During mitosis the nuclear envelope is not broken down and the mitotic spindle is formed outside the nucleus. It is still a matter of debate how dinoflagellates achieve chromatin condensation.

Until recently, dinoflagellates were believed to lack histones and thus a typical nucleosomal chromatin organization [13], and that instead basic histone-like proteins (HLPs) [14] may play an important role. This view has changed since Lin et al. [15] discovered all four core nucleosomal histones (H2A.X, H2B, H3, and H4), along with histone deacetylase and nucleosome assembly protein during a spliced leader-based metatranscriptomic analysis. This may indicate the presence of a functional nucleosome-like machinery. Alternatively, these histones may be involved in the regulation of gene expression. It is likely that in earlier studies histones and other nucleosome-associated proteins were not detected because they are expressed at very low levels. This also fits with the dinoflagellate chromatin protein∶DNA ratio of 1∶10 which is too low to explain packaging of the entire genome. The low amounts of histones and the presence of peripheral DNA loops are probably due to a secondary loss and gain of the corresponding genes in free-living dinoflagellates, as parasitic species and a free-living basal species, *Oxyrrhis marina*, possess histones, whereas DNA loops are not found [16]. Taken together, the way dinoflagellates facilitate their chromatin condensation/decondensation is still enigmatic.

Recently, another peculiarity of dinoflagellate genomes was discovered - in all species studied so far, an invariant 22 bp trans-spliced leader (SL) was found at the 5′end of full-length mRNAs [17], [18], [19] indicating a trans-splicing mechanism, which is currently described only for a few organisms (e.g. trypanosomes, nematodes and hydra). The exact function and extent of this mechanism in dinoflagellates is unknown but may involve, in analogy to trans-splicing in other organisms, resolution of polycistronic pre-mRNAs [20] and a role in mRNA stability or translatability [21], [22]. Lidie et al. [17] suggested that trans-splicing might help dinoflagellates to compensate for their atypically large genomes that are devoid of stereotypical transcriptional regulators. Furthermore, Slamovits and Keeling [23] identified relict SL-sequences of diverse dinoflagellates in tandem repeats in cDNAs, as well as in the genomic DNA, and hence postulated that genes can cycle continuously between DNA and RNA. The relict tandemly repeated SL sequences are truncated after nucleotide 7 of the canonical SL corresponding to an AG dinucleotide splicing site and are located directly downstream of the canonical sequence. This pattern indicates that expressed and trans-spliced genes are reverse-transcribed and reintegrated into the genome where they then supposedly undergo the next cycle of expression, trans-splicing and reintegration. Taken together, dinoflagellates exhibit an unusual genome organization and gene regulation and transcription which give rise to many speculations about the evolution of potentially novel mechanisms to achieve this.

Dinoflagellate EST surveys and sequencing of target genes indicate that many genes have high copy numbers and are arranged in tandem. Bachvaroff and Place [24] analysed genomic sequences for 47 genes from *Amphidinium carterae* and postulated two general categories of genes in dinoflagellates - a highly expressed class of tandem repeats and a less highly expressed class of intron-rich genes.

The organellar genomes of dinoflagellates are highly reduced compared to their counterparts in other eukaryotes and exhibit extraordinary organizations and regulation of gene expression. Only very few proteins - cytochrome c oxidase subunit 1 (*cox1*) and subunit 3 (*cox3*), and cytochrome b (*cytb*) - as well as fragmented rRNAs were found to be encoded in the mitochondrial genome, and these together with intergenic regions underwent duplication, fragmentation and scrambling giving rise to an overall complex organization [25]. The gene content of the dinoflagellate chloroplast has also been dramatically reduced, with a large-scale transfer of genes to the nucleus [26]. Only a handful of genes, encoding subunits of Photosystems I and II, the cytochrome b6f complex, ATP synthase, RNAs and tRNAs, reside in the chloroplast genome, which is partitioned into ‘minicircles’ [27], [28].

Investigation of genomic sequence data of dinoflagellates is a challenge, particularly due to their huge genomes. Examination of dinoflagellate genomes has therefore typically been limited to particular genes (randomly found or amplified based on previously known sequence information) or snapshots of mainly non-coding sequences [29]. The large knowledge gap about the overall nature of dinoflagellate genomes provokes a number of fundamental questions: are genes or other recognizable sequence elements clustered or evenly spread throughout the genome? Are there zones with different characteristics (e.g. isochors)? How many types of repeats are present in the genome and do they exhibit any special features? Do non-coding repeated sequences play a role in chromatin condensation mechanisms of dinoflagellates?

In this study, we established a cDNA library to gain a general overview of the transcriptome of the spirolide-producing *A. ostenfeldii* strain AOSH2. Our EST set was compared to data from other *Alexandrium* species (*A. minutum*, *A. tamarense*, and *A. catenella*) regarding stop codon usage, polyadenylation signals, as well as truncated repeated SL sequences. Moreover, fosmid- and BAC libraries were constructed and end-sequenced for *A. ostenfeldii* to uncover gene content and to investigate the distribution of repetitive sequences, simple repeats, RNA pseudogenes, and transposable elements (TEs). Finally, we obtained a glimpse of local sequence organization from completely sequencing a randomly selected fosmid clone.

## Results

### Transcriptomic characterization of *A. ostenfeldii*

A total of 12,287 ESTs from strain AOSH2 were assembled into 8,438 unique sequences (855 tentative consensus sequences, TCs, and 7,583 singlets) by the clustering and assembling tool TGICL with an minimum overlap length of 40 bp, at least 95% sequence similarity and a maximum number of mismatches of 30 bp. The average length of the unique sequences was 639 bp and the global GC content of the ESTs was 53.1%. The GC content of mitochondrial transcripts (*cytb*, *cox1* and *cox3*) was 40.3%, of rRNA transcripts 42.0% and of the nuclear transcripts 55.2%.

Table 1 lists the 24 most highly represented transcripts. Several of the most abundant transcripts encoded genes that are encoded in the mitochondrial genome in all dinoflagellates studied in this regard, i.e. *cytb* (265 ESTs), *cox1* (249 ESTs), *cox3* (81 ESTs), and transcripts encoding both *cytb* and *cox3* (43 ESTs). Other highly represented transcripts included those for 28S rRNA, peridinin-chlorophyll a-binding protein (*pcp*), potassium channel, luciferin-binding protein, heat shock proteins 70 (*HSP70*) and 90 (*HSP90*), ubiquitin, actin, elongation factors *EF1A* and *EF-G*, and three hypothetical proteins without unequivocal similarity to sequences in the databases. Highly significant for dinoflagellates, putative histone-like protein (*hlp*) transcripts were found in our library, although they were not highly represented (one TC comprising 3 ESTs).

**Table 1:** The 24 most represented transcripts in the *A. ostenfeldii* EST library.

no. of TCs	no. of ESTs	% of total ESTs	Annotation
31	355	2.89	28S rRNA
37	265	2.16	cytochrome b
21	249	2.03	cytochrome c oxidase subunit 1
5	185	1.51	peridinin-chlorophyll a-binding protein
2	185	1.51	potassium channel
15	81	0.66	cytochrome c oxidase subunit 3
7	43	0.35	cytochrome b and cytochrome c oxidase subunit 3
7	29	0.24	luciferin-binding protein
2	25	0.20	18S rRNA
8	22	0.18	calcium-dependent protein kinase
2	21	0.17	ubiquitin
3	19	0.15	heat shock protein 70
4	16	0.13	alcohol dehydrogenase, zinc-containing
1	15	0.12	elongation factor EF1A
1	15	0.12	hypothetical protein
2	15	0.12	voltage-dependent T-type calcium channel
5	14	0.11	actin
3	14	0.11	heat shock protein 90
6	14	0.11	serine/threonine-protein kinase
1	13	0.11	elongation factor EF-G
3	13	0.11	ribonucleoside-diphosphate reductase small chain
1	12	0.10	hypothetical protein
1	12	0.10	hypothetical protein
4	12	0.10	Pentatricopeptide (PPR) repeat-containing protein

All TCs (tentative consensus sequences = clustered and assembled ESTs using TIGR clustering-algorithm, and cap3) having the same annotation were added and the number of ESTs as well as the percentage of the total number of ESTs is shown. Annotations were allocated when at least one BLAST hit to one of the databases had an e-value<10^−30^.

**Figure 1:**
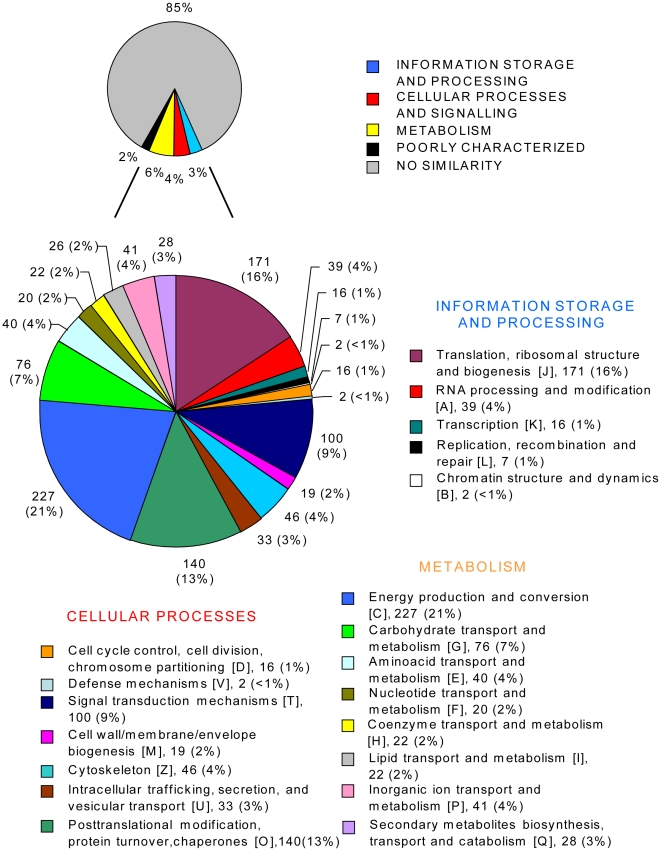
KOG category distribution of unique sequences of the *A. ostenfeldii* cDNA library. Numbers of unique sequences and their share of total number are shown. The category “poorly characterized” includes “general function prediction only (R)” and “function unknown (S)”.

For functional classification the unique sequences were annotated according to KOGs, and revealed affinities representing all of the major functional categories (Figure 1). Nevertheless, a remarkably high number (85%) of unique sequences displayed no similarity to any sequence in the databases. A total of 6% were genes assignable to general metabolism, 4% to cellular processes and signalling and 3% to information storage and processing. Of the annotatable sequences (Figure 1, large pie chart) most fell into the categories “energy production and conversion (C)” (21%), “translation, ribosomal structure and biogenesis (J)” (16%), and “posttranslational modification, protein turnover, chaperones (O)” (13%). All EST sequences have been deposited in GenBank (GenBank: HO652585–663459).

### Spliced-leader (SL)-sequences at *Alexandrium* EST 5′ ends

**Figure 2:**
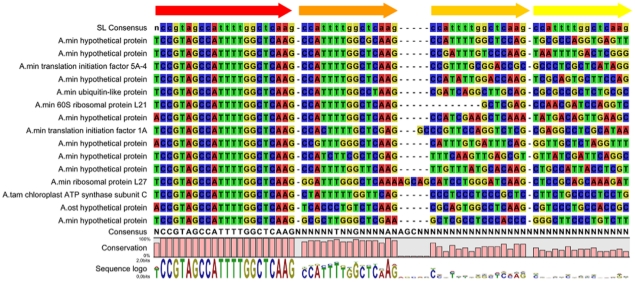
Alignment of ESTs of *Alexandrium* spp. showing spliced leader (SL) and relict SL-repeats with different conservation levels. A reference SL consensus sequence (first sequence in lower case) is shown. The generated consensus sequence, sequence conservation and sequence logo are represented under the alignment. See File S1 for the alignment of the complete SL-containing EST set.

Screening of all available *Alexandrium* ESTs (*A. ostenfeldii*, *A. minutum*, *A. catenella* and *A. tamarense* retrieved from Genbank) for SL-sequences revealed 238 unique sequences containing the full motif. These were pooled from 325 total ESTs with full motif. The highest number of unique SL-containing transcripts came from *A. tamarense* (131), followed by *A. ostenfeldii* (96). Only five and one unique SL-containing transcripts were found in *A. tamarense* and *A. catenella*, respectively, due to the fact that the sequences derived from NCBI were not raw data. In 61 (26%) of these ESTs a single repeated truncated SL was present with identities ranging from 60 to 100% with respect to the canonical SL sequence. Examples are shown in Figure 2. A total of 11 ESTs contained a second truncated SL repeat with identities of 60 to 100%, and a third truncated SL repeat was present in five ESTs (identities 50 to 63%). A fourth SL repeat was identified in one EST with 53% identity. Furthermore, in *A. ostenfeldii*, *A. minutum* and *A. catenella* some ESTs containing an unusually long 5′ UTR were found.

The partial alignment of the different SL sequence patterns (Figure 2) clearly shows the decrease in conservation due to insertions, deletions and mutations in the downstream relict SL-sequences. Whereas the first repeat could be easily identified in some of the sequences, sequence similarity progressively degenerated in the following repeats. File S1 shows the alignment of the complete set of SL containing transcripts. We found no SL-containing mitochondrial or rRNA transcripts, as expected.

### Stop codon utilization and polyadenylation signals in *Alexandrium* species

Examination of *A. ostenfeldii* and *A. minutum* EST contigs for which we considered open reading frame prediction highly reliable yielded stop codon predictions for 97 sequences from *A. ostenfeldii* and 165 from *A. minutum*. The different stop codons were utilized in remarkably similar proportions in *A. ostenfeldii* and *A. minutum* (Table 2), with TGA being most frequent (76.3% and 77.0%, respectively). Searching the same sequences for conserved patterns in the 3′-most 60 bp preceding the Poly-A stretch failed to identify any potential common polyadenylation signal sequence motif.

**Table 2:** Distribution of stop codons in *A. ostenfeldii* and *A. minutum*.

species	stop codon	no. of stop codons	% of total stop codons	number of total stop codon predictions
*A. ostenfeldii*	TAA	7	7.2	
	TAG	16	16.5	
	TGA	74	76.3	97
*A. minutum*	TAA	13	7.9	
	TAG	25	15.2	
	TGA	127	77.0	165

### Features of *A. ostenfeldii* genomic sequences

The terminal bi-directional sequencing of about 5,700 fosmid clones yielded 4,709 usable reads after quality inspection. To test for repetitiveness, reads were assembled and formed 3,084 contigs (File S2). The terminal bi-directional sequencing of about 384 BAC clones resulted in 558 usable reads after quality inspection. Assembling yielded 445 contigs (File S3). Due to the low overall sequence coverage, assembling the reads in order to reconstruct longer contiguous segments of genomic DNA was unrealistic. We used the method of sequence assembly for a different purpose, in order to identify non-unique (repetitive) sequence stretches. For the fosmid and BAC read assembly the STADEN package (http://staden.sourceforge.net) was used with a minimum overlap of 100 bases and at least 90% identity. This way we took into account moderate sequence divergences and sequencing errors. Given the low coverage with large clone end sequences each non-singlet contig represents sequences, which are present at least twice in the whole genome. All genomic contig sequences have been deposited in GenBank (GenBank:HN262719–266253).

**Figure 3:**
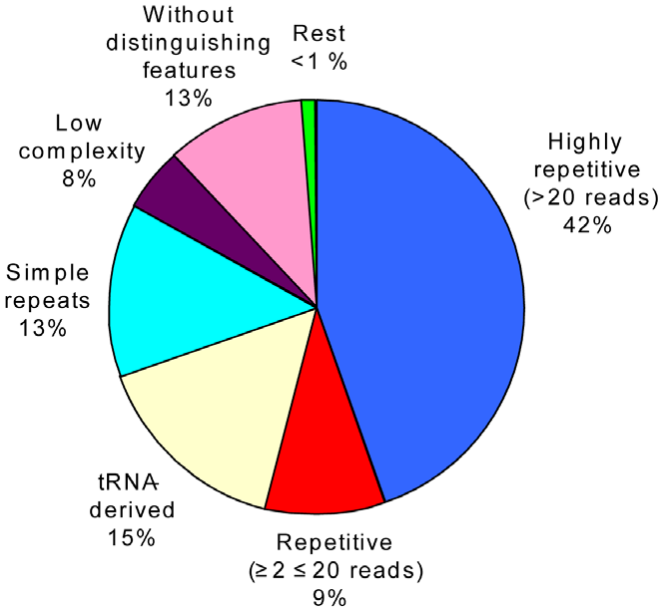
Distribution of features of *A. ostenfeldii* genomic sequences illustrated with combined BAC and fosmid data. “Rest” includes genes (16), viral sequence, SINE, LINE, LTR retrotransposons, DNA transposons, RNA pseudogenes (snRNA, scRNA, rRNA), and satellites. Repetitive sequences were identified by contig generation within each library and BLASTn between the two libraries. Transposons were identified as described in the text. The remaining features were determined by RepeatMasker analysis. The category “Without distinguishing features” includes all single reads with no BLAST or RepeatMasker hit.

**Figure 4:**
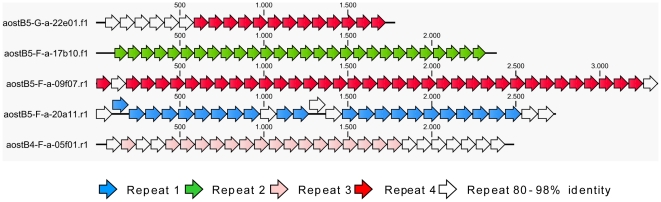
Large tandem arrays within the five most repetitive fosmid contigs. Contigs comprise 98 reads (aostB4-F-a-05f01.r1), 338 reads (aostB5-F-a-20a11.r1 and one BAC read), 277 reads (aostB5-F-a-09f07.r1), 71 reads (aostB5-G-a-22e01.f1) and 313 reads (aostB5-F-a-17b10.f1).

The degree of repetitiveness between the fosmid and the BAC libraries determined by BLASTn justified the combined analysis and characterization of the fosmid and BAC sequences as one data set. Note that different features were often detected within one contig and thus some contigs were counted more than once to create the overview pie chart (Figure 3). Table 3 provides both percentages referring to Figure 3 and referring to the actual total sequence (6,21 Mbp). In the following, proportions of different features correspond to Figure 3. The high repetitiveness of fosmid and BAC sequences was remarkable (Figure 3, Table 3, Files S2 and S3). The overall GC-content was 60%. A total of 1,926 reads were repeated at least twice, corresponding to 51% of the total sequence data. Of these reads 1,204 (42%) were highly repetitive (>20 reads). More detailed analysis of the five most repetitive sequences (contigs comprising the most reads) showed that each contig contained large tandem arrays (Figure 4) with repeats of 97 bp (repeat 1), 79 bp (repeat 2), 88 bp (repeat 3), and 88 bp (repeat 4) length (Table 4). The tandem arrays of repeats 1, 3 and 4 were interspersed with repeats of 88–98% identity relative to the major repeat type present (see also File S4). Repeat 3 and 4 share a 60 bp region with 93% identity (Table 4, underlined). The tandem repeats did not show any hit to RepeatMasker [30] or Repbase (using CENSOR [31]) nor (after translation into all six reading frames) to the Pfam databases [32]. For each contig the number of repeats (including those with 88–98% identity) was multiplied by the repeat length and number of reads to estimate their fraction in the total sequence. The largest fraction was found for repeat 4 (28.11% of total sequence). Strikingly, together these tandem arrays comprised about 58% of our *A. ostenfeldii* genomic DNA sample. Moreover, when the five contigs were individually blasted against all other fosmid and BAC contigs, several positive matches (90–100% identity) were found to regions containing similar tandem arrays with varying similarity between the repeats and to the query (data not shown).

**Table 3:** Distribution of features of *A. ostenfeldii* genomic sequences.

	no. of reads	total bp	% of total bp (pie chart)	% of total bp (actual)
**genes**	16	12366	0.14	0.20
**virus**	1	610	0.01	0.01
**highly repetitive (>20 reads)**	1204	3712171	41.72	59.79
**repetitive (2 reads at least)**	722	802600	9.02	12.93
**LTR retrotransposons**	8	5709	0.06	0.09
**DNA transposons**	5	3567	0.04	0.06
**snRNA**	22	27131	0.30	0.44
**scRNA**	1	773	0.01	0.01
**rRNA**	4	3503	0.04	0.06
**tRNA-derived**	299	1301387	14.62	20.96
**satellites**	5	4852	0.05	0.08
**simple repeats**	1452	1145806	12.88	18.45
**low complexity**	739	677401	7.61	10.91
**with distinguishing features**	3012	5016515	56.37	80.80
**without distinguishing features**	2871	1200887	13.49	19.34
**100% actual (clones)**	5883	6208876		100.00
**100% pie chart**		8898763	100.00	

BAC and fosmid library data are combined. The proportions of the different features refer to the full length contigs that include the feature. As different features were often detected within one contig, some contigs were counted more than once and, thus, the total number of bp used to create the pie chart in Figure 3 is an overestimation. This table provides both percentages referring to Figure 3 and referring to the actual total sequence.

**Table 4:** Repeats 1–4 that form large tandem arrays within the five most repetitive fosmid contigs.

**Repeat 1**
AATAGCGCTTGAGGTGCGCGCGCGTTTCTTCCGAATGCCCAAGACGGTT TTGGCGTTTTCAGTGGCGGAGCCTCGCGGCGCCCCGAAGCCCGGAGCG
Length: 97 bp
GC-content: 64%
∼14.79% of total genome
**Repeat 2**
CCGCTCAGCCGCCTGAGCGAGCAATTCCTCGAGCGAGTCCTCCATGGTA TGTCCACAAAGTGGCTACGCCCTCTTTGGC
Length: 79 bp
GC-content: 61%
∼11.15% of total genome
**Repeat 3**
GTCCGGAGCCAGAAGCAAGAGAAGCAAT**TGAAATTGACCGCAAGAGTGC CCTTTTACTGGCTCCGGCCTAAAGCTTGTCGCGAGCGAG**
Length: 88 bp
GC-content: 55%
∼3.75% of total genome
**Repeat 4**
**TGAAATTGACCGCAATAGTGCCCTTTTTGTGGCTCCGGCCTAAAGCGTG TCGCGAGCGAG**GTCCGGAGCCAGAAGCAGGAGAAGCAAT
Length: 88 bp
GC-content: 56%
∼28.11% of total genome

Repeat 3 and 4 share a 60 bp region with 93% identity (bold). Together the tandem arrays involving repeats 1–4 comprise at least 58% of *A. ostenfeldii* genomic DNA.

To further verify that the tandem repeat structures we found within the five most repetitive fosmid contigs are also present in the single fosmid clones and are not an artifact of the contig assembly, we subjected to fragments of about three representative clones to restriction digest using enzymes that were selected to cut once per repeat motif (File S5). We found that 05f01 plasmids were almost completely digested 100 bp, corresponding well to the size of the repeat motifs. The 22e01 and 17b10 plasmids were also digested to 100 bp fragments, although a portion of 17b10 was apparently not digested at all and 22e01 showed several fragments of 1100 bp to 400 bp length. Overall our results suggest that almost the entire plasmid 05f01 consists of the described tandem repeats, whereas over 50% of the plasmids 17b10 and 22e01 contain tandem repeats. Since the clone inserts were at least 25 kb in size our results strongly support that the tandem arrays (Figure 4) are not an artefact of contig assembly. Regardless of the exact local organization, the four described non-unique sequences seem to comprise a substantial fraction of the *A. ostenfeldii* genome.

RepeatMasker analysis revealed that the *A. ostenfeldii* genomic sequence data contained 15% tRNA-derived sequences, 13% simple repeats (1–6 bp long), and 8% low complexity regions (Table 3, File S6). Less than 1% was made up of genes (16 identified), viral sequences, LINE, SINE, LTR retrotransposons, DNA transposons, other RNA pseudogenes (snRNA, scRNA, rRNA), and satellites. A total of 13% of the sequence did not contain any distinguishing features; in other words, they represented all single reads with no BLAST or RepeatMasker hit. Within the simple repeats, (CACG)n, (CATG)n, and (CGTG)n were the most abundant, comprising 1.77%, 1.48% and 1.47% of the total sequences, respectively. Within the low complexity regions, GA-rich (2.26%), GC-rich (1.84%), and C-rich (1.08%) were the most prevalent. Among the tRNA-derived sequences, tRNA-Pro-CCG (6.71%), tRNA-Ala-GCA (4.44%) and tRNA-Phe-TTY (2.19%) were most abundant. Within the sequences similar to TEs (0.37% of total sequence), those with hits to human LINEs were predominant (0.21% of total sequence), Table 5 and File S6.

**Table 5:** RepeatMasker identification of putative transposon sequence stretches of the *A. ostenfeldii* genomic sequence.

contig name	contig length	no. of reads	transposon name	transposon class	bp with transposon
**BACs**					
AOSH-G-b-02b09.r1	676	1	ERE1_EH	Interspersed repeat	82
AOSH-G-b-06f04.f1	711	1	HAL1-2a_MD	LINE	115
**Fosmid**					
aostB5-F-a-16h02.f1	826	1	hAT-50_HM	DNA transposon	46
			Copia-38_BD-I	LTR retrotransposon	34
aostB5-G-a-22e06.f1	398	1	DNA-12N_Sbi	DNA transposon	88
aostB5-F-a-10g05.r1	828	1	GYPSY3-I_CB	LTR retrotransposon	83
			L1-2_Fc	LINE	102
aostB5-F-a-30g10.f1	785	1	hAT-11_SM	DNA transposon	72
aostB5-F-a-25c02.f1	779	1	hAT-Charlie, MER58A	DNA transposon	100
			EnSpm6_SB	DNA transposon	119
aostB4-F-a-07a10.r1	771	1	HERV17-int	LTR retrotransposon	138
aostB4-G-a-02c10.r1	843	1	HERV17-int	LTR retrotransposon	115
aostB5-F-a-09d04.f1	631	1	HERV17-int	LTR retrotransposon	229
aostB4-F-a-07f02.r1	392	1	MER4-int	LTR retrotransposon	49
aostB5-F-a-10d05.f1	740	1	MER4-int	LTR retrotransposon	54
aostB1-F-a-05e08.f1	678	1	MLT2B2	LTR retrotransposon	96
aostB4-G-a-03a11.f1	717	1	HAL1-2a_MD	LINE	156
aostB4-G-a-03g03.f1	723	1	HAL1-2a_MD	LINE	275
aostB5-F-a-06d03.r1	667	1	HAL1-2a_MD	LINE	271
aostB5-F-a-30b09.f1	862	1	HAL1-2a_MD	LINE	111
aostB5-G-a-22c07.f1	619	1	HAL1-2a_MD	LINE	324
aostB5-G-a-22h05.r1	811	1	HAL1-2a_MD	LINE	165
aostB1-F-a-05d05.f1	505	1	HAL1-3A_ME	LINE	57
aostB1-F-a-05d05.r1	821	1	HAL1-3A_ME	LINE	240
aostB4-F-a-06b07.r1	761	1	HAL1-3A_ME	LINE	168
aostB5-F-a-08c05.r1	1157	1	HAL1-3A_ME	LINE	56
aostB5-F-a-10h04.r1	528	1	HAL1-3A_ME	LINE	44
aostB4-F-a-01e10.r1	861	1	L1M3e	LINE	226
aostB5-F-a-02a05.r1	738	1	L1M3e	LINE	711
aostB5-G-a-22d04.f1	304	1	L1M3e	LINE	274
aostB5-G-a-22d04.r1	534	1	L1M3e	LINE	364
aostB4-G-a-02e08.r1	837	1	L1MC4	LINE	268
			L1MEc	LINE	437
			AluSc5	SINE	130
aostB4-G-a-02e08.f1	852	1	L1ME3E	LINE	336
			AluSz6	SINE	85
			L1ME3F	LINE	271
aostB5-F-a-08b01.r1	773	1	AluSz	SINE	299
aostB5-F-a-08b01.f1	768	1	AluSz6	SINE	127

RepeatMasker was applied using default values (human repeat database).

Annotation by BLASTx against the Swiss-Prot [33] and Genpept (GenBank Gene Products) database revealed 16 sequences that encoded fragments of genes (12,366 bp, 0.2% of total bp). These sequences were annotated as fatty acid desaturase, actin, phospholipase D, ras guanine nucleotide exchange factor, phosphatidylinositol 3-kinase 2, asparaginyl-tRNA synthetase (*asnS*), ribosomal large subunit pseudouridine synthase F (*rluF*), and ubiquitin, as well as two uncharacterized proteins and two without any hit. Four sequences were identified to encode mitochondrial proteins, namely NADH-quinone oxidoreductase chain M, NADH-quinone oxidoreductase chain G, *cox1* and *cytb* (see Files S2 and S3). A total of 318 sequences (144,671 bp, 2.43% of total bp) were of low complexity and had low similarity to known proteins but these were not considered to be valid genes.

In summary, 16 gene fragments were discovered in 6.21 Mbp of *A. ostenfeldii* sequence. Approximately half (51%) of the genomic DNA displayed complex repeats, with simple repeats and tRNA also highly abundant, while only 13% of the sequence was without any distinguishing features. BLASTn of all *A. ostenfeldii* genomic sequences against *H. triquetra* genomic data yielded no conserved motifs, except a few short regions of 71–78 bp (data not shown).

Shotgun sequencing of one complete fosmid clone yielded 1,669 reads comprising an average of 675 bases per read of high quality data. Of these reads, 1,090 were assembled into an insert sequence of 34,308 bp (GenBank: HQ437322), whereas the rest comprised clone vector sequences. A first analysis showed that this fosmid contained no identifiable gene fragment. RepeatMasker analysis of the sequence (File S7) revealed that most of the sequence contained simple repeats (5,326 bp, 15.53% of total sequence), 7.31% (2,506 bp) contained low complexity regions, and 0.89% (306 bp) contained sequence with hit to a LINE motif (HAL1-2a MD). Among the low complexity regions, most were made up of C-rich sequence (6.19% of total sequence). Simple repeats (1–6 bp) sometimes occurred in tandem. Within the simple repeats, the most abundant were (CTG)n, (CCA)n, (CG)n, (TGG)n and (ATGGTG)n, representing 1.92%, 1.77%, 1.19%, 1.02% and 1.02%, respectively. Larger repeats (up to 14 bp) were also abundant and sometimes present in tandem repeats. A region of 775 bp matched with one of the fosmid contigs (aostB4-G-a-02c03.f1, e-value 0.00) and a region of 399 bp was highly similar to fosmid contig aostB5-F-a-16c07.f1 (e-value 3×10^−179^). These were counted as long repeats (3.42% of total sequence).

In summary, a total of 34,308 bp continuous fosmid sequence contained mainly simple repeats and low complexity regions. The sequence also contained two longer regions matching with other fosmid clones.

## Discussion

### Transcriptomic characterization of *A. ostenfeldii*

The high number (8,438) of unique sequences in our *A. ostenfeldii* EST library possibly represents an overestimation of the true number of unique genes because some may represent alternatively spliced transcripts, which cannot be properly aligned. Yang et al. [34] found putative different splice variants in about 3% of the contigs of an EST library of *A. minutum*. Another reason for the high number of unique sequences comes from the general trend of dinoflagellate genes to form large families. Several other dinoflagellate EST projects showed similar library complexity, including those that were the source of EST data used for the comparison of *Alexandrium* species (e.g. [34], [35], [36], [37]).

The global EST GC content (53.1%) found in *A. ostenfeldii* lies within the expected range for dinoflagellates (e.g. [36], [38]), as well as the low percentage of annotatable ESTs by BLAST (15% of unique sequences) [35], [38], the latter reflecting large evolutionary distances from well-annotated organisms.

The highest frequency genes found in our *A. ostenfeldii* library (see Table 1) were mostly typical for dinoflagellate EST surveys [35], [36], [37], [38]. One of the TCs was similar to a putative histone-like protein (*hlp*) and seems to be dinoflagellate-specific, as it displayed BLAST hits only to other dinoflagellates, with the best hit to the distantly related heterotrophic species *Crypthecodinium cohnii* (e-value 1.6×10^−22^, Blast2n vs. nt).

Surprisingly, all ESTs coding for ribosomal RNAs contained polyA stretches. We assume, that most likely, these polyA stretches are the result of reverse transcription by the polyT primer annealing to a short complementary stretch in the rRNA.

The dinoflagellate mitochondrial genome is likely subject to a high rate of intramolecular recombination. Evidence for this comes from the presence of multiple copies of protein (*cox1*, *cox3*, *cytb*) and rRNA genes that occur in different genomic contexts and are often fragmented [25]. In conformity with this, we observed a substantial diversity of mitochondrial transcripts (*cox1*, *cox3* and *cytb*) clustered into multiple TCs in *A. ostenfeldii*. The highly abundant mitochondrial *cox3*-*cytb* chimeric transcripts (43 ESTs forming 7 TCs) seem to be a prevalent form in dinoflagellates, as similar combined transcripts were found in *Durinska baltica* (*cox1*-*cytb*-*cox1*) [39] and *Gonyaulax polyedra* (now *Lingulodinium polyedrum*, *cox3*-*cytb*) [40], and are likely transcribed from chimeric genes as were amplified from mtDNA of *Durinska baltica* [39], *L. polyedrum* [40], and *Oxyrrhis marina* (*cytb*-*cox3*) [41].

### SL-sequences at *Alexandrium* EST 5′ ends

Recycling of mRNAs seems to be a feature typical for dinoflagellates [23]. This mechanism could have only been detected because dinoflagellates apply SL trans-splicing of perhaps all nuclear encoded genes, whereby a conserved 22-nt SL sequence is spliced onto the 5′end of transcripts. The recycling mechanism starts with a mature SL-containing mRNA that is reverse-transcribed and reintegrated into the genome at a new location where it increases gene copy number. Upon expression of this new gene, a second SL is trans-spliced at the splice-acceptor site (AG) of the first SL, thereby truncating it. With reintegration of this processed mRNA a new cycle starts and depending on the number of cycles relict SL sequences will accumulate. Within the genome they have time to mutate, resulting in decreasing sequence similarity towards 3′end.

We searched a subset of 238 ESTs likely representing full-length genes as indicated by functional SL sequences for relict SL-sequences. Relict SL-sequences were detectable in 25% of the ESTs containing the full SL motif, comparable to the 22% found among various other free-living marine dinoflagellates [23]. These findings do not obviate the possibility that a higher percentage of the genes have been recycled because their relict SL-sequences could have mutated beyond recognition or trans-splicing could have excised them. The reintegration of mRNAs into the genome indicates once more the high plasticity of the dinoflagellate genome. The mRNA recycling may be the underlying reason for unresolved problems in gene expression studies because such recycling constitutes an additional variant of post-transcriptional processes. Maintaining this mechanism may confer evolutionary advantages upon dinoflagellates. Gene duplication, for example, facilitates neo-functionalization of genes. This plasticity of the genome is beneficial for horizontal gene transfer, which could provide dinoflagellates with new advantageous cell functions in highly dynamic aquatic environments. In addition, as pointed out by Slamovits and Keeling [23], assuming the addition of the SL at the mRNA level is essential, the SL sequence itself favours its own presence at the 5′ end of genes due to the conservation of two AG splice-acceptor sites.

Moreover, results of Bachvaroff and Place [24] indicate two general categories of genes in dinoflagellates: a highly expressed tandem repeat class and an intron-rich lower expressed class. This fits well to the concept of recycling of mRNAs, as a highly expressed gene would undergo more recycling and thereby would lose more intron sequence. Conversely, it is reasonable to assume that mRNAs carrying relict SL sequences are transcribed from genes belonging to a higher expressed gene family than mRNAs without them. It would be very interesting to test a possible correlation with a larger sample size of full-length transcripts and a broad taxon sampling. The combination of the two mechanisms, SL trans-splicing and mRNA recycling, appears to be unique among eukaryotes. On the other hand, the recycling in other SL *trans*-splicing species might have escaped recognition due to less conserved SL sequences, as several SL families within single species and sequence variation within these families are commonly found, e.g. in mertensiid ctenophores, hexactinellid sponges and amphipod and copepod crustaceans [42].

### Stop codon utilization and polyadenylation signals in *Alexandrium* species

Stop codon usage by *A. ostenfeldii* and *A. minutum* corresponds well with that in *A. tamarense* [35], where the stop codon TGA was heavily favoured (79.8%) over the stop codons TAG (14.6%) and TAA (5.6%). Similar results were obtained for *A. catenella* [37] with TGA in 72.7%, TAG in 20.7% and TAA in 6.5% of the stop codons examined. As this feature was found in all four species and over a wide range of genes, stop codon distribution seems to be a stable characteristic in *Alexandrium*.

Some limited codon usage data of other dinoflagellates was available through the Codon Usage Database (GenBank Release 160.0, June 2007), i.e. *Karlodinium micrum*: 26 total stop codons, 30.77% TGA, 26.92% TAG, 42.31% TAA, *Heterocapsa triquetra*: 42 total stop codons, 90.48% TGA, 2.38% TAG, 7.14% TAA, *Symbiodinium* sp.: 44 total stop codons, 68.18% TGA, 11.36% TAG, 20.45% TAA (File S8). These data suggest a trend to favour TGA in *Heterocapsa* and *Symbiodinium* similar to *Alexandrium* but not in *Karlodinium* and no clear trend is apparent for the other two stop codons, possibly due to the small sample size. Regarding other members of chromalveolates the overall picture gained from the data through the Codon Usage Database, considering *Phaeodactylum tricornutum*, *Plasmodium falciparum*, *Cryptosporidium parvum*, *Perkinsus marinus*, *Phytophthora sojae*, and *Emiliania huxleyi*, indicates a common preference for stop codon TAA over TAG whereas TGA is least frequent (File S8). This fits to the findings of Sun et al. [43] that TAA usage is overrepresented in the lower eukaryotes, whereas TGA usage is overrepresented in the higher eukaryotes, though they did not state which species were included in the analysis. Thus, dinoflagellates once again represent an exception to the rule by using TGA as the main stop codon (except *K. micrum*). Moreover, stop codon frequencies within the genus *Alexandrium* are more similar than among less closely related dinoflagellates. Clearly, to verify this first impression a larger sample size has to be analyzed.

The reasons for stop codon bias, as for codon bias in general, are highly debated for all organisms but the GC content in coding regions could provide some explanation because a strong bias towards G and C at the third position in amino acid codons was also found in *A. tamarense* [35] and *A. catenella* [37].

A dinoflagellate polyadenylation signal present only in genomic sequences was recently identified from intergenic spacer sequences [24]. The exact polyadenylation site in *Amphidinium carterae* contained the sequence AAAAG/C; the mRNA polyA tail presumably started after the fourth A. This motif was confirmed in *Karenia veneficum* and *Lingulodinium polyedrum* in the same study. We examined the *A. ostenfeldii* and *A. minutum* EST libraries for additional conserved motifs in the 60 bp 5′ of the PolyA regions. However, HMM searches failed to identify overrepresented 5–9 bp motifs; this was not affected by restricting the search region to the 30 bp closest to the PolyA tail. Together with the results for *A. tamarense*, for which no polyadenylation signal could be identified using n-mer searches and a Gibb's sampler method [35], this reinforces the suggestion that dinoflagellate polyadenylation signals are located in the 3′ sequence part removed during mRNA processing [24].

### Features of *A. ostenfeldii* genomic sequences

The genomic sequence of *A. ostenfeldii* seems to be highly structured. This stands in sharp contrast to findings of the only other reported dinoflagellate GSS; the *H. triquetra* genomic sequence [29] showed no distinguishing features in 90% of the sample and only 5.2% comprised complex repeats. This most likely reflects the difference in sample size as we analysed about 27 times more sequence data (6.21 Mbp in total) than was done for *H. triquetra* (233.05 kbp). Stochastically, an increase in sample size enhances the chance to find repeated sequences. It has to be noted that still our genomic sample represents a minor fraction of the complete genome. Assuming a DNA content of 114.9 pg/cell as measured for *A. ostenfeldii* by flow cytometry [44] and the general formula: genome size (bp) = (0.921×10^9^)×DNA content (pg), our total sample would constitute 0.0059% of the genome.

The gene-coding percentage of the sample in *A. ostenfeldii*, 0.2% (calculated based on 16 detected genes), was the same as found in *H. triquetra* (one putative gene, 0.2% of total sample). Hou and Lin [45] used completely sequenced and annotated genomes from phylogenetically diverse lineages of eukaryotes and non-eukaryotes to investigate the relationship between gene content and genome size using regression analyses. This was done with a view to predict the gene content of non-sequenced dinoflagellate genomes. They predicted a gene-coding percentage of 1.8% for the smallest documented dinoflagellate genome (*Symbiodinium spp.*, 3×10^6^ kbp in total) and 0.05% for the largest (*Prorocentrum micans*, 245×10^6^ kbp). In terms of estimated genome size and gene-coding percentage, *A. ostenfeldii* (genome size: 105.3×10^6^ kbp [44]) and *H. triquetra* (genome size: 18.6–23.6×10^6^ kbp [45]) lie within this range. Other lower eukaryotes have smaller genomes and accordingly higher gene-coding percentages. Comparable to dinoflagellate gene-coding percentages are that of a few higher eukaryotes only, e.g. *Gallus gallus* (3.1%, genome size: 1031880.1 kbp), *Homo sapiens* (1.2%, genome size: 3080436 kbp), *Mus musculus* (2.0%, genome size: 2644094 kbp), *Rattus norvegicus* (1.7%, genome size: 2718897.3 kbp), but their genome sizes are still considerably smaller than that of dinoflagellates; see File S1 [45].

Two of the mitochondrial genes detected in *A. ostenfeldii*, *cytb* and *cox1*, have been found in all dinoflagellate mitochondrial genomes studied so far. Thus, it is possible that these genomic sequences were derived from the mtDNA portion of the total DNA used for fosmid and BAC library generation. Yet, our data do not allow us to discern between the nuclear or mitochondrial genome origin of these sequences. Interestingly, we found six putative SL RNA genes (data not shown) in the genomic sequences, which demonstrates the value of our genomic data to be used for deeper analyses of the SL trans-splicing mechanism in *A. ostenfeldii* in the future.

Another noteworthy finding is the presence of 15% (see Fig. 4) tRNA-related sequences in *A. ostenfeldii*, as opposed to not a single identified RNA-related sequence in the *H. triquetra* study [29]. These authors applied the same detection method (RepeatMasker) as we did for *A. ostenfeldii*, but might have used different threshold values for detection. Simple repeats and low complexity regions were also more abundant in *A. ostenfeldii* than in *H. triquetra*.

We assume that our findings reflect the essential characteristics of the nuclear genome. Our results show that the *A. ostenfeldii* genome is characterized by a high degree of sequence repetitiveness on multiple levels (repeated large tandem arrays, repeat 1–4, similarity between repeat 3 and 4, other simple repeats). The vast majority of the genome appears to consist of large tandem arrays, which can be divided into at least five categories according to their repeat structure (Figure 4). Together they comprise at least 58% of the total sequence. The large amount of repetitive sequence in *A. ostenfeldii* genomic DNA accords with the results of several physical studies of the genome of the heterotrophic dinoflagellate *C. cohnii*, involving hydroxylapatite binding, digestion with S1 nuclease and restriction enzymes, reassociation kinetics, and/or electron microscopy [46], [47], [48]. The combined results suggested that about half of the genome is composed of repeated sequences. Similar results were found for the dinoflagellate *Woloszynskia bostoniensis* [49].

The detection of large tandem repeats in *A. ostenfeldii* raises the question of whether or not they represent a common feature of dinoflagellates. In this case, it would be interesting to assess if the repeated units show any similarity among different lineages or species and how they are distributed within the permanently condensed chromosomes. However, no larger conserved sequences between *A. ostenfeldii* and *H. triquetra* were detected. This apparent lack of conservation may be an artefact – due to limited sequence information available for *H. triquetra* – but more likely indicates that simple repeats are species specific. Large tandem repeats, as found in centromeres, are often regions of tightly packed chromatin (heterochromatin) and thus it is conceivable that tandem repeats contribute to the overall compactness of dinoflagellate chromosomes, either by sequence complementarity alone or by involvement of structural proteins that recognize these sequence parts. The hypothesis that tandem repeats alone may cause heterochromatin formation was already formulated in 1944 [50]. In dinoflagellates, the protein∶DNA mass ratio is 1∶10 (as opposed to 1∶1 in other eukaryotes); we speculate therefore that in dinoflagellates large tandem arrays are scattered within the chromosomes and that condensation is mainly achieved through sequence complementarity.

Some workers have hypothesized specific sequences to mediate initiation and termination of heterochromatin propagation along a chromosome [51]. Other studies found evidence for repeat-induced gene silencing (RIGS) (reviewed by [52]). Transgenic experiments often involve the insertion of multi-copy tandem arrays of transgenes at specific sites. The expression of these transgenes was shown to be suppressed depending on repeat number in the brassicacean plant *Arabidopsis* (e.g. [53]) and in mice [54], and heterochromatin formation was observed at these sites. Moreover, RIGS is suggested to provide another genome defence mechanism against, e.g., transposons, as tandem repeat arrays generated by TEs would promote heterochromatin formation and thus prevent their own propagation.

The high degree of repetitiveness at large scales, represented by conserved tandem arrays, as well as at smaller scales (repeat 1–4, similarity between repeat 3 and 4, simple repeats) could be interpreted as component of a condensation/decondensation mechanism that provides spatial flexibility. More precisely, the chromatin could alter its structure (due to condensation and decondensation to form peripheral loops where gene expression takes place), while concurrently maintaining a compact form through binding of complementary DNA of various lengths and spatial flexibility due to putative uniformly distributed repeats. This could potentially explain the permanently condensed form of dinoflagellate chromatin. The putative nucleosome-like machinery [15] may be at work at the chromatin periphery for fine tuning gene expression. However, it is unlikely that the small amount of histones play a major part in maintaining overall condensation.

Several mechanisms have been proposed for the production of tandem repeats. These include replication slippage [55], unequal crossing over [56] and also TE activity. Overall, sequences showing hits to TEs were found in 0.37% of our sequence sample (see Table 5). In general TEs encounter little selection pressure and thus many dinoflagellate species-specific TEs may be too divergent from known TEs to be detected by our approach. Although the proportion of TEs in our sample was relatively small, the large tandem repeats could be the result of TE activity. It is generally accepted that differential amount of repetitive DNA account for a major fraction of eukaryotic genome size variation [57], [58] and that TEs play an important role in genomic plasticity and restructuring. How the coding part of the dinoflagellate genome is organized, remains an open question. Are genes scattered throughout the genome or are they organized in islands? Moustafa et al. [59] argue that among microbial eukaryotes, dinoflagellates exhibit the highest gene number. However, we found only a very low number of genes in our GSS. We take this as indirect evidence for the gene island model. However, this hypothesis needs to be further substantiated by additional studies on dinoflagellate genomes.

In conclusion, our results confirm that *A. ostenfeldii* as well as three other *Alexandrium* species are typical dinoflagellates regarding lack of polyadenylation signals within mature mRNAs and SL trans-splicing of nuclear encoded genes. The presence of relict SL sequences in multiple transcripts adds to the growing body of evidence that mRNA recycling is a universal feature in dinoflagellates. It provides one possible explanation why dinoflagellate genomes are such flexible in incorporating foreign DNA, most apparent in the massive transfer of chloroplast genes to the nucleus.

Except for a single attempt [29], GSSs of dinoflagellates were avoided in the past due to potential problems posed by very large genome sizes and associated difficulty to encounter coding parts, bacterial contamination, and unusual DNA bases. Nevertheless, we were able to provide a data set of 6.21 Mbp of *A. ostenfeldii* sequence that for the first time allowed insights into the strikingly high amount of repetitive sequence of this dinoflagellate. These repetitive sequences are mainly constituted of large tandem arrays of only four repeat types. Our findings provide an important step towards better understanding of dinoflagellate genome organization and might serve as a basis to test hypothesis regarding chromatin condensation and decondensation mechanisms with a low amount of basic nuclear proteins, a matter of debate for many years.

## Methods

### Dinoflagellate strain isolation and culture

*Alexandrium ostenfeldii* AOSH2, clonally isolated from Ship Harbour, Nova Scotia, Canada, was used for generation of the cDNA library. The isolate was grown in 1 L borosilicate glass flasks on modified K medium [60] prepared with filtered North Sea seawater (about 32.5 psu). Cultures were rendered axenic by multi-antibiotic treatment (50 µg ml^−1^ ampicillin, 3.3 µg ml^−1^ gentamycin, 25 µg ml^−1^ streptomycin, 1 µg ml^−1^ chloramphenicol and 10 µg ml^−1^ Ciprofloxacin) for about 10 days (from inoculation until harvesting). Dinoflagellate cells were also subjected to five washing steps with sterile seawater and harvested by filtration through 8-µm pore size TETP membrane filters (Millipore) to yield an axenic sample. Prior to dinoflagellate culture harvest, small sub-samples were stained with acridine orange and checked by epifluorescence microscopy for bacterial contamination. Only axenic cultures were used for downstream applications.

For cDNA library production strain AOSH2 was grown under eight different environmental treatments of various light, temperature and nutrient conditions to increase the diversity of mRNA species. One group was grown on a light∶dark photocycle of 14∶10 h at 15°C at a photon flux density of 90 µmol m^−2^ s^−1^, considered to be the optimal light regime (N. Jaeckisch, unpublished observations). These cultures were harvested at 06:00 (start of light phase) and at 00:00 (start of dark phase). Another group of cultures was grown under nitrogen deprivation (−N) or under low N conditions (50% of normal K medium) and harvested in the middle of exponential growth phase. Other cultures were harvested after 3 days maintained at 10°C, 5°C, in constant darkness, under low light intensity (30 µmol m^−2^ s^−1^,) and after 1 h exposure to high light (600 µmol m^−2^ s^−1^).

### Construction of cDNA libraries

The EST libraries of *A. ostenfeldii* AOSH2 was prepared from total RNA extracted separately from each environmental treatment mentioned above in Sigma TRI-Reagent (Sigma-Aldrich, Steinheim, Germany) following the manufacturer's protocol. RNA was purified with RNeasy (Qiagen, Hilden, Germany) including an on-column DNase treatment to eliminate contaminating genomic DNA. RNA quality and quantity were analyzed by UV-spectrometry at 230, 260 and 280 nm with a NanoDrop ND.1000 Spectrophotometer (PeqLab Biotechnologie, Erlangen, Germany) and with an Agilent 2100 Bioanalyzer (Agilent Technologies, Böblingen, Germany). The RNA corresponding to the nine different treatments was pooled to a total of 40.8 µg and further processed by Vertis Biotechnology (Freising, Germany). Poly A^+^ RNA was purified from this total RNA and full-length enriched cDNA synthesis was performed. An oligo(dT)-linker primer was used for first-strand synthesis. The resulting cDNA was amplified with 18 LA-PCR cycles to generate a non-normalized cDNA library.

A normalized library was also constructed from one cycle of cDNA denaturation and reassociation. Reassociated ds-cDNA was separated from the remaining ss-cDNA, the normalized cDNA, by passing through a hydroxylapatite column. Subsequently, the ss-cDNA was amplified with 12 LA-PCR cycles. Non-normalized and normalized cDNA were directionally ligated into the plasmid vector pBS II sk+. The non-normalized cDNA was cloned into XL1-Blue MRF' electro-competent cells, resulting in a total of 860,000 clones, and the normalized cDNA was cloned into TransforMAX™EC100™-T1^R^ electro-competent cells (Epicentre, Madison, WI, USA), yielding 930,000 clones. Both cDNA libraries were sequenced from the 3′ end. During analysis we discovered that normalization had failed and thus decided to compile the data sets of both libraries into one common reference set to have the broadest basis for the following analyses. Sequence data were analysed by the SAMS 2.0 platform (T. Bekel, Bioinformatics Resource Facility, CeBiTec, Bielefeld, Germany), imposing a length and quality e-value cut-off of 100 bp minimum and 10^−10^–10^−13^ maximum, respectively. After vector and quality clipping, 12,287 ESTs were further analyzed. Within the SAMS pipeline, clustering and assembly of the ESTs is carried out using the clustering and assembly tool TIGR Gene Indices Clustering (TGICL, www.tigr.org/tdb/tgi/software) which results in Tentative Consensus Sequences (TCs) and singletons. The “clustering” phase is intended to partition the input data set into smaller groups of sequences (clusters) by pairwise alignments (using megablast) that have stringent similarity and are potentially coming from the same longer original sequence. In the assembly phase each such cluster is sent to the assembly program (cap3), which attempts the multiple alignment of the sequences in the cluster and creates one or more contigs (consensus sequences). The minimum overlap length is 40 bp and 95% of required similarity and maximum number of mismatches of 30 bp. The goal is the reconstruction of full mRNA sequences, ideally one sequence per cluster, representing a single gene. Redundancy helps to correct putative sequencing errors.

All ESTs were assembled to form 855 TCs with 7,583 singlets remaining, resulting in 8,438 unique sequences. Automated BLAST runs against various sequence databases (Blast2n vs. nt, Blast2x vs. KEGG, Blast2x vs. KOG, Blast2x vs. SP, Blast2x vs. nr), and a motif search using InterPro, were performed by the SAMS 2.0 software. Annotations of all TCs and subsets of the singlet annotations were inspected manually. Annotations were given the prefix “similar to” if hits in the databases had e-values of 10^−4^ to 10^−10^ and “putative” if e-values were <10^−10^. No prefix means hits had E values≤10^−30^. To gain an overview of the cellular functions represented by the ESTs the unique sequences (TCs and singlets) were sorted according to KOGs (clusters of orthologous groups) for eukaryotic complete genomes.

For *in silico* comparison among *Alexandrium* species, EST sequences of *A. tamarense* [35] and *A. catenella* [37] were retrieved from the database of the National Center for Biotechnology Information (NCBI). The EST sequences of *A. minutum*, also accessed from NCBI, were generated within our research group [34].

### Examination of SL sequences in *Alexandrium*

ESTs of all four *Alexandrium* species were examined for the presence of SL-sequence. Hits containing the full 22 bp trans-spliced leader were aligned using the MAFFT Multiple Sequence Alignment algorithm implemented in the Jalview alignment editor [61]. Redundant ESTs were removed and the remaining unique sequences were trimmed upstream of the SL-motif. Sequences were subsequently searched downstream of the SL-motif for occurrence of the truncated SL-sequence starting with 100% down to 50% sequence identity with the CLC Main Workbench package 5.1 [62].

### Stop codon utilization and polyadenylation signals

We checked for stop codon utilization in *Alexandrium* by examining the stop codons of *A. ostenfeldii* and *A. minutum* transcripts. To ensure reliability of the sequence comparisons, we included only sequences producing SwissProt BLAST hits of 1e^−50^ or better. Reading frame predictions were based on OrfPredictor [63] outputs. Nucleotide sequences and in-frame translations were manually compared using Geneious Pro version 4.6.4/4.6.5 [64].

Based on BLAST similarity data, some sequences were identified as plastid-associated sequences that are known to be encoded on minicircles in at least one dinoflagellate species [27] (e.g. *psaA*, *psaB*, *psbA*-*E*), or as one of the genes encoded in the highly reduced mitochondrial genome [27] (*cox1*, *cox3*, *cytb*). These putatively organellar transcripts were excluded from the data set, as well as frameshift-containing and 3′-incomplete sequences. Presumed stop codons were examined manually. Where possible, stop codon predictions for *A. ostenfeldii* categories were based on at least 3 EST sequences. The same sequences were used to search for polyadenylation signals. Sequences were truncated to 60 bp 5′ of the beginning of the PolyA regions, and searched using the Hidden Markov Model pattern search as implemented in CLC Main Workbench 5.1. [62]. *A. tamarense* and *A. catenella* ESTs were not subjected to this analysis because the ESTs retrieved from NCBI did not contain poly(A)-tails.

### Fosmid library generation

Freshly harvested axenic cells of *A. ostenfeldii* strain AOSH2 were mixed with TE buffer (20 mM EDTA, 10 mM Tris*Cl) and dropped into liquid nitrogen using a pipette. Each sample comprised cells of 800 ml of dense culture in logarithmic growth phase. The resulting pearls were stored at −70°C until further processing. The pearls were ground together with sterile sand with a mortar and pestle until the sample was completely pulverized. The sample was kept frozen during processing with liquid nitrogen. The powder was incubated at 45°C for 1.5 h in a lysis buffer (15 ml per sample) with gentle agitation, following a user-developed protocol for Genomic-tip (Qiagen, Hilden, Germany). The lysis buffer contained 20 mM EDTA, 10 mM Tris*Cl, 0.5 mg ml^−1^ cellulase, 1% Triton X-100, 500 mM guanidine-HCl and 200 mM NaCl. The mixture was supplemented with DNase-free RNase A (20 µg ml^−1^) and incubated for 2 h at 50°C with gentle agitation. Samples were centrifuged for 20 min at 15.000×g to pellet insoluble debris. The clarified lysate was transferred to Qiagen Genomic-tip 100/G equilibrated with QBT buffer and high molecular weight DNA was isolated following the manufacturer's protocol. DNA quality was checked with a NanoDrop ND.1000 Spectrophotometer (PeqLab Biotechnologie, Erlangen, Germany). DNA size was assessed with a Chef Mapper™ XA pulsed field electrophoresis system (BioRad, Munich, Germany). DNA fragments of 36–45 kbp length were purified from low-melting point agarose. For library generation the CopyControl Fosmid Library Production Kit (Epicentre, Hess. Oldendorf, Germany) was applied following the manufacturer's instructions. Fragments of about 36 kbp were cloned into the pCC1FOS™ vector (Epicentre, Hess. Oldendorf, Germany) resulting in 5,700 clones that were terminal bi-directionally sequenced. One randomly selected fosmid clone was additionally completely shotgun sequenced and assembled into one contiguous sequence.

### BAC library generation

Genomic DNA was prepared by embedding *Alexandrium ostenfeldii* cells in agarose strings, subsequently lysed with proteinase K and collagenase, three times for 24 h. Clean plugs were partially digested by HindIII (40 units) for 4 min, and the reaction was stopped on ice with 7 µl 0.5 M EDTA. DNA fragments were separated according to size by pulsed-field gel electrophoresis (Chef Mapper™, BioRad, Munich, Germany) and electroeluted from the gel. DNA fragments were then ligated to pINDIGO BAC5–HindIII cloning ready (Epicentre Technologies, Hess. Oldendorf, Germany) at a molar ratio of insert to vector of 10∶1. The ligation product was mixed with EC100 electrocompetent cells (Epicentre Technologies) and electroporated on a Gene Pulser Xcell (BioRad, Munich, Germany). After 20 h at 37°C on LB chloramphenicol (12.5 g ml^−1^) plates, recombinant colonies were picked into 96-well microtitre plates containing 60 µl of 2YT medium plus 5% glycerol and 12.5 g ml^−1^ chloramphenicol, grown for 18 h at 37°C, duplicated and stored at 80°C. The resulting 384 BAC library clones had inserts between ∼50 kb and ∼150 kb. The insert ends were sequenced using vector based oligos as primers.

### Sequence analysis of fosmid and BAC library

To test for repetitiveness fosmid and BAC end sequences were assembled separately into contigs for each library using the STADEN package (http://staden.sourceforge.net) with a minimum overlap of 100 bases and at least 90% identity. These contigs were annotated based on BLAST hits against Swissprot and Genepept and also subjected to RepeatMasker [30] analysis. The repetitiveness between the fosmid and BAC libraries was determined by BLASTn. The most repetitive sequences (contigs comprising the most reads) were inspected in more detail using GeneQuest (Lasergene; DNAStar Inc., Madison, WI, USA), Pfam [32] and CENSOR [31]. Because species-specific transposons are usually not detected by RepeatMasker, *A. ostenfeldii* transposons in the genomic data were identified as follows: BAC and fosmid sequences were blasted directly against all transposon, transposase and integrase entries in NCBI by local BLAST (CLC). Moreover, the ESTs of *A. ostenfeldii* (this study), *A. minutum* (NCBI), *A. catenella* (NCBI), and *A. catenella* (NCBI) were scanned for transposon-related sequences and hits were blasted against BAC and fosmid sequences. The proportions of the different features were calculated referring to full length contigs that include the feature. When different features were found within the same contig, the full length contig was counted for each feature. The fosmid and BAC sequences were blasted against all genomic data retrieved from NCBI for the marine dinoflagellate *H. triquetra* to identify putative conserved motifs or longer repeats shared between the two species. The completely assembled fosmid clone was also analysed by RepeatMasker to detect simple repeats and conserved motifs, and blasted against the terminally sequenced fosmid and BAC clones to reveal putative conserved regions.

To verify that the tandem repeat structure we found within the five most repetitive fosmid contigs are also present in the single fosmid clones and are not an artefact of the contig assembly, we subjected three representative clones to restriction digest. Clones 17b10, 5f01 and 22e01 corresponded to the contigs having the same designation (Figure 4) and 0.5 µg of their plasmid DNA was digested with restriction enzyme XhoI (17b10) and AciI (5f01 and 22e01) at 37°C for 1 h and the enzymes were subsequently heat inactivated at 65°C for 20 min. Restriction enzymes (New England Biolabs) were chosen to cut once per repeat motif. A total of 5 µl of the samples were loaded into a 1% agarose gel containing ethidium bromide and gel electrophoresis was performed at 90 V for 50 min.

## Supporting Information

File S1 **The complete SL-containing EST sets of** ***A. ostenfeldii*** **,** ***A. minutum*** **,** ***A. catenella*** **and** ***A. tamarense*** **.**
(FAS)

File S2 **Fosmid library contig list with annotations.**
(XLS)

File S3 **BAC library contig list with annotations.**
(XLS)

File S4 **Alignment of repeat motif 1, 3, and 4.**
(XLS)

File S5 **Gel electrophoresis of restriction digested fosmid clones.**
(XLS)

File S6 **Overview of RepeatMasker results of** ***A. ostenfeldii*** **genomic sequences (BAC and fosmid data combined).**
(TIF)

File S7 **Overview of RepeatMasker results of one completely sequenced fosmid clone.**
(TIF)

File S8 **Overview of stop codon usages in dinoflgellate and other chromalveolates.**
(XLS)
