# Supplementary figures and images for: Comparative Genomic and Transcriptomic Characterization of the Toxigenic Marine Dinoflagellate Alexandrium ostenfeldii
Source: PLoS One. 2011 Dec 2;6(12):e28012. doi: 10.1371/journal.pone.0028012 (PMC3229502; doi:10.1371/journal.pone.0028012)

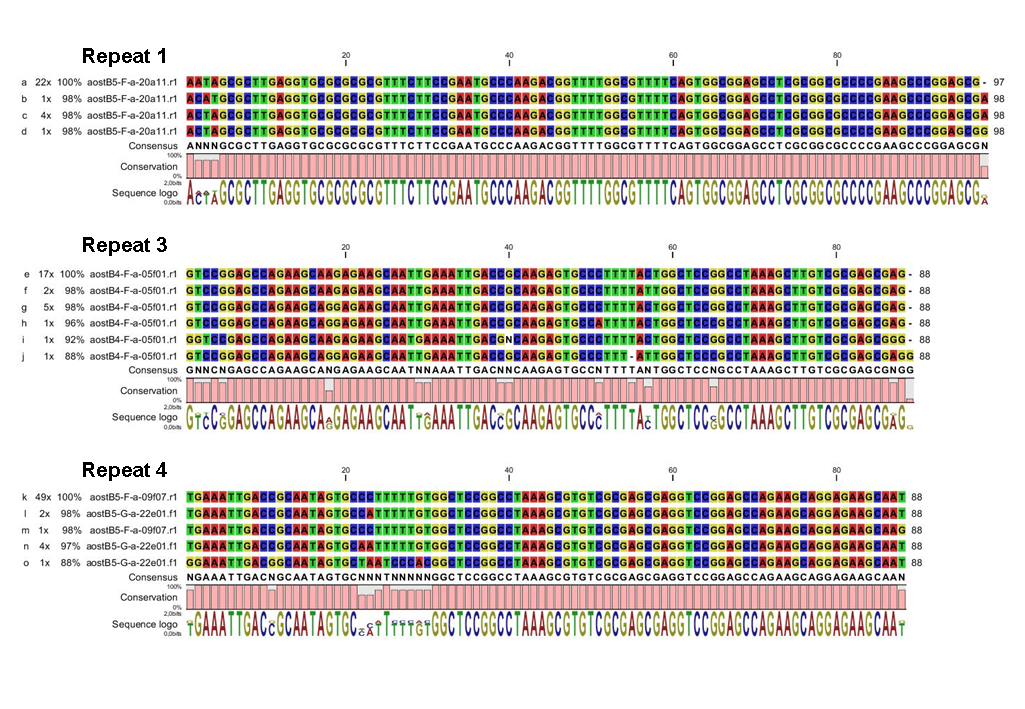

Supplement: File S6 — Overview of RepeatMasker results of A. ostenfeldii genomic sequences (BAC and fosmid data combined). (TIF) [file pone.0028012.s006.tif]

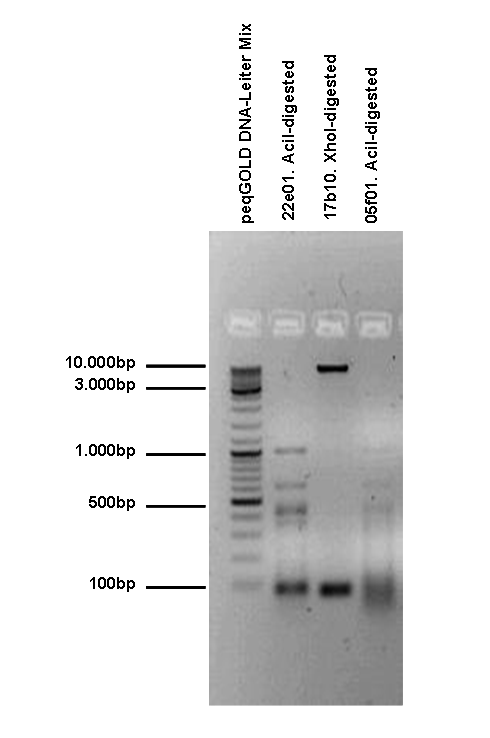

Supplement: File S7 — Overview of RepeatMasker results of one completely sequenced fosmid clone. (TIF) [file pone.0028012.s007.tif]
